# Cilostazol Induces PGI_2_ Production via Activation of the Downstream Epac-1/Rap1 Signaling Cascade to Increase Intracellular Calcium by PLCε and to Activate p44/42 MAPK in Human Aortic Endothelial Cells

**DOI:** 10.1371/journal.pone.0132835

**Published:** 2015-07-16

**Authors:** Ayako Hashimoto, Michinori Tanaka, Satoshi Takeda, Hideki Ito, Keisuke Nagano

**Affiliations:** 1 First Institute of New Drug Discovery, Otsuka Pharmaceutical Co., Ltd., Tokushima-shi, Tokushima, Japan; 2 Medical Chemistry Research Institute, Otsuka Pharmaceutical Co., Ltd. Tokushima-shi, Tokushima, Japan; 3 Qs'Research Institute, Otsuka Pharmaceutical Co., Ltd. Tokushima-shi, Tokushima, Japan; BloodCenter of Wisconsin, UNITED STATES

## Abstract

**Background:**

Cilostazol, a selective phosphodiesterase 3 (PDE3) inhibitor, is known as an anti-platelet drug and acts directly on platelets. Cilostazol has been shown to exhibit vascular protection in ischemic diseases. Although vascular endothelium-derived prostaglandin I_2_ (PGI_2_) plays an important role in vascular protection, it is unknown whether cilostazol directly stimulates PGI_2_ synthesis in endothelial cells. Here, we elucidate the mechanism of cilostazol-induced PGI_2_ stimulation in endothelial cells.

**Methods and Results:**

Human aortic endothelial cells (HAECs) were stimulated with cilostazol and PGI_2_ accumulation in the culture media was measured. Cilostazol increased PGI_2_ synthesis via the arachidonic acid pathway. Cilostazol-induced intracellular calcium also promoted PGI_2_ synthesis via the inositol 1,4,5-trisphosphate receptor. Using RNAi, silencing of PDE3B abolished the induction effect of cilostazol on PGI_2_ synthesis and intracellular cAMP accumulation. Inhibition of the exchange protein, which was directly activated by cyclic AMP 1 (Epac-1) and its downstream signal the Ras-like small GTPase (Rap-1), abolished cilostazol-induced PGI_2_ synthesis, but this did not take place via protein kinase A (PKA). Inhibition of downstream signaling, such as mitogen-activated protein kinase (MAPK), phosphoinositide 3-kinase (PI3K) γ, and phospholipase C (PLC) ε, suppressed cilostazol-induced PGI_2_ synthesis.

**Conclusions:**

The PDE3/Epac-1/Rap-1 signaling pathway plays an important role in cilostazol-induced PGI_2_ synthesis. Namely, stimulation of HAECs with cilostazol induces intracellular calcium elevation via the Rap-1/PLCε/IP3 pathway, along with MAPK activation via direct activation by Epac-1/Rap-1 and indirect activation by Epac-1/Rap-1/PI3Kγ, resulting in synergistically induced PGI_2_ synthesis.

## Introduction

Cilostazol [6-[4-(1-cyclohexyl-1*H*-tetrazol-5-yl) butyloxy]-3,4-dihydroquinolin-2-(1*H*)-one] is a selective phosphodiesterase 3 (PDE3) inhibitor, which has been shown to prevent platelet aggregation and peripheral vasodilation [1]. The PDE3 family, known for catalyzing cyclic adenosine monophosphate (cAMP), comprises two members, PDE3A and PDE3B, which exhibit different expression patterns. PDE3A is mainly present in the heart, platelets, vascular smooth muscles, and oocytes, whereas PDE3B is mainly found in adipocytes, hepatocytes, and spermatocytes [2]. Cilostazol similarly inhibits both PDE3A and PDE3B, with IC_50_ values of 0.20 and 0.38 μM, respectively [3]. Cilostazol is the only medication with a class I indication approved by the Food and Drug Administration (FDA) for intermittent claudication [4]. Recent reports have demonstrated that cilostazol also exerts pleiotropic effects [5], due to unknown mechanisms, independent of its direct effects on platelets and smooth muscle cells. Vascular protection strategies, defined as augmentation of endothelial function, have focused on and proved effective in preventing ischemic vascular events [6]. In healthy vessels, endothelial cells produce the vasoactive hormones, nitric oxide (NO), and prostacyclin (PGI_2_) [7]. NO and PGI_2_ are regarded as key mediators of vascular protection and play important roles in the modulation of vascular tone, as well as anti-inflammatory and anti-thrombotic properties [8]. The loss or attenuation of NO and PGI_2_ production is an early marker of endothelial dysfunction found in many ischemic diseases [9]. Both are coreleased by agonist-stimulated endothelial cells via intracellular calcium elevation, indicating that increased intracellular calcium activates endothelial nitric oxide synthase (eNOS) for NO synthesis and phospholipase A2 (PLA_2_) to liberate arachidonic acid for PGI_2_ production [7]. Various in vivo and in vitro studies have demonstrated that cilostazol exhibits vascular protection via eNOS activation, leading to beneficial impacts on ischemic diseases, including myocardial infarction [10], stroke [11], and limb ischemia [12]. Compared with the large volume of evidence for NO-involved vascular protection by cilostazol, the association between cilostazol and PGI_2_ production remains unclear. However, it is reasonable to speculate that cilostazol activates PGI_2_ production, as well as NO production. Igawa et al [13]. were the first to show the involvement of PGI_2_ in cilostazol-exerted anti-platelet action, and that endothelial cells potentiated the inhibitory effect of cilostazol on platelet aggregation, which was antagonized by a cyclooxygenase (COX) inhibitor. However, PGI_2_ synthesis in endothelial cells was not measured in their study, thus the goal of the present study was to address this question by examining whether and how cilostazol stimulates PGI_2_ production in endothelial cells.

## Methods

### Materials

Cilostazol was synthesized by Otsuka Pharmaceutical Co., Ltd (Tokyo, Japan). N(6),2'-O-dibutyryladenosine 3':5' cyclic monophosphate (dbcAMP), cilostamide, milrinone, rolipram, zaprinast, erythro-9-(2-Hydroxy-3-nonyl)adenine hydrochloride (EHNA), ionomycin, AS605240 (selective PI3Kγ inhibitor), PD98059 (selective ERK inhibitor), 2-aminoethyl diphenylborinate (2-APB), and indomethacin (COX inhibitor) were purchased from Sigma-Aldrich (St. Louis, MO, USA). 6-Bn-cAMP (PKA-selective cAMP analogue) and 8-pCPT-2'-O-Me-cAMP (Epac-1-selective cAMP analog; 007) were obtained from Alexis Biochemicals (San Diego, CA, USA). Myristoylated cell-permeable PKA inhibitor peptide sequence (14–22) amide was obtained from Calbiochem/Merck (Darmstadt, Germany). O,O'-Bis(2-aminophenyl) ethyleneglycol-N,N,N',N'-tetraacetic acid and tetraacetoxymethyl ester (BAPTA-AM) was from Dojindo Laboratories (Kumamoto, Japan). For western blot analysis, the primary antibodies used were specific to Epac-1 (Abcam, Cambridge, MA, USA), phospho-PDK1, PDK-1, phospho-MAPK (p44/42), MAPK, Rap-1A/B, phospho-Akt (ser473), Akt (Cell Signaling Technology, Danvers, MA, USA), phospho-phospholipase A_2_ (PLA_2_), PLCε (Santa Cruz Biotechnology, Santa Cruz, CA, USA), and regulatory subunit of PKA type II (RII) β (Upstate/EMD Millipore Corporation, Billerica, MA, USA). HRP-conjugated secondary antibodies were from Cell Signaling Technology. For positive and negative control for PDE3A and 3B, total protein lysates of normal adult human adipose tissue and artery were purchased from BioChain Institute, Inc. (Newark, NJ, USA). For immunofluorescence histochemistry, the primary antibodies used were anti-PDE3B, anti-PDE3A (Abcam), and anti-VE Cadherin (R&D Systems Minneapolis, MN, USA). The secondary antibodies, Alexa fluor488- and 568-conjugated antibodies, were from Life Technologies, Inc. (Carlsbad, CA, USA). siRNAs against *PDE3A* and *PDE3B* (Hs.591150 and Hs.445711, respectively), and control siRNA were purchased from Life Technologies, Inc. siRNA against *Epac-1* (sc-41700), *Rap-1* (sc-38554), and *PLCε* (sc-44024) were purchased from Santa Cruz Biotechnology, Inc. For Biacore analysis, human recombinant PI3Kγ protein was from OriGene Technologies, Inc. Biotin-labeled Epac-1-binding PDE3B peptide (Met-1 to Glu-25; MRRDERDAKAMRSLQPPDGAGSPPE-K-biotin-NH2) and biotin-labeled PI3Kγ-binding PDE3B peptide (Met-1 to Glu-25; MRRDERDAKAMRSLQPPDGAGSPPE-K-biotin-NH2) were purchased from Toray Research Center, Inc. (Tokyo, Japan). Unlabeled PDE3B-binding Epac-1 peptide-1 (Thr-218 to His-242: Ac-ELLLEAMGPDSSAHDPTETFLLDFL-NH2), and PDE3B-binding Epac-1 peptide-2 (Glu-398 to Lys-422: Ac-TVALRKPPGQRTDEELDLIFEELLH-NH2) were synthesized by Otsuka Pharmaceutical Co., Ltd.

### Cell culture

Human aortic endothelial cells (HAECs; PromoCell GmbH, Heidelberg, Germany) were cultured in 5% CO_2_ at 37°C in 100-mm culture dishes containing endothelial cell growth medium (EGM-2) supplemented with 2% fetal bovine serum, 10 pg/mL epidermal growth factor, 1 μg/mL hydrocortisone, 12 μg/mL bovine brain extract, and 0.1% gentamicin sulfate and amphotericin-B (PromoCell GmbH). Cells from passages 4–8 were used for all experiments.

### Cellular cAMP level

HAECs were plated in 96-well culture plates at a density of 5 × 10^3^ cells/well and cultured overnight. After 15 min incubation with cilostazol, cells were lysed with lysis reagent (RPN225, Amersham Biosciences, Buckinghamshire, UK) cAMP concentration was determined using a cAMP EIA kit (Amersham Biosciences,) according to the manufacturer’s instructions.

### siRNA (small interfering RNA) transfection

HAECs were plated in 96-well culture plates at a density of 1 × 10^3^ cells/well. After overnight incubation, HAECs were transfected with the indicated siRNAs (1.2 pmol/well) with Lipofectamine RNAiMAX Reagent (Life Technologies, Inc.) according to the manufacturer’s instructions. After 4 h incubation, the transfection medium was replaced with EGM-2 complete medium and knockdown was assessed at 48 h. Knockdown of target proteins were verified by western blotting.

### PGI_2_ level

HAECs were plated in 24-well culture plates at a density of 5 × 10^4^ cells/well and cultured overnight. Culture media were replaced with 250 μL of EGM-2 containing test drugs and incubated for 1 h. Supernatants were collected and stored at –80°C until further analysis. PGI_2_ was assessed as 6-keto prostaglandin F_1α_ (6-keto PGF_1α_) using the 6-keto PGF_1α_ enzyme immunoassay (EIA) kit (Cayman Chemical, Michigan, USA) according to the manufacturer’s instructions. Optical density was measured at 405 nm using a microplate reader (Soft max, Molecular Devices, Sunnyvale, CA, USA). Results are expressed as 6-keto PGF_1α_ concentration (pg/mL).

### Intracellular calcium concentration

HAECs were plated on 8-well chamber glass slides at a density of 1 × 10^4^ cells/well and cultured overnight. Then, cells were loaded with 2 μM fluo-4 AM (Molecular Probes, Life Technologies), a fluorescent calcium indicator, for 15 min. Cells were pretreated with or without BAPTA-AM (100 μM) or 2-APB for 15 min, and then stimulated with test drugs. After treatment with cilostazol, cells were stimulated with 1 mM ionomycin to obtain a maximal response. The absorption shift of fluo-4 AM upon binding of Ca^2+^ was determined by scanning the excitation light at 480 nm. Fluorescent images of individual cells were analyzed every 2 s with a confocal laser scanning microscope (TCS-SP5, Leica Microsystems GmbH, Wetzlar, Germany).

### Inositol 1,4,5-trisphosphate (IP3) concentration

HAECs were plated in 96-well culture plates at a density of 1 × 10^4^ cells/well and cultured overnight. Culture media were replaced with 250 μL of EGM-2 containing test drugs and incubated for 1 h. Supernatants were collected and stored at –80°C until further analysis. IP3 was assessed using human inositol 1,4,5-trisphosphate, IP3 ELISA Kit (Cusabio, Wuhan, China) according to the manufacturer’s instructions. Optical density was measured at 450 nm using a microplate reader (Soft max, Molecular Devices). Results are expressed as IP3 concentration (pg/mL).

### Immunofluorescence histochemistry

HAECs grown on 8-well chamber glass slides at a density of 1 × 10^4^ cells/well were washed with PBS on ice and fixed with 4% paraformaldehyde for 30 min. Cells were washed with PBS, permeabilized, and blocked with 0.5% blocking reagent (PerkinElmer Inc., Waltham, MA, USA) in PBS for 30 min at room temperature. Cells were incubated overnight at 4°C with primary antibodies against PDE3B (1:200 dilution) or PDE3A (1:200 dilution) in the presence of anti-VE Cadherin (1:100 dilution). The primary antibodies were detected by incubation with Alexa fluor-conjugated secondary antibodies (1:500) for 60 min at room temperature. Cells were washed with PBS and mounted with Fluorescence Mounting Medium (Fluoromount/Plus, Dako North America, Inc., Carpinteria, CA, USA). Fluorescent images were analyzed with a confocal laser-scanning microscope (TCS-SP5) equipped with a 64× water immersion objective.

### Western blot analysis

HAECs were starved overnight in endothelial cell basal medium (EBM-2; PromoCell GmbH). After treatment with reagents for 1 h, cells were lysed with RIPA buffer (50 mM Tris-HCl, pH 8, 150 mM NaCl, 1% Nonidet P40, 0.5% sodium deoxycholate, and 0.1% SDS) containing protease and phosphatase inhibitors (100× Halt Protease and Phosphatase Inhibitor Cocktail, Thermo Fisher Scientific Inc., Waltham, MA, USA). Protein concentrations were measured using the Bio-Rad DC protein assay (Bio-Rad, Hercules, CA, USA). Cell lysates (25 μg/lane) were subjected to 10% SDS-PAGE and transferred to a polyvinylidene difluoride (PVDF) membrane (Trans-Blot Turbo Mini PVDF Transfer Packs, Bio-Rad). After blocking with 5% skim milk in Tris-buffered saline, the membranes were incubated overnight at 4°C with primary antibodies against PDE3A, PDE3B, Epac-1, PKA RIIβ (1:200 dilution), phospho-PDK1, PDK-1, phospho-Akt, Akt, phospho-MAPK, MAPK, Rap-1A/B (1:250), phospho-PLA_2_, or PLA_2_ (1:100), followed by incubation with secondary antibodies (1:1000 dilution). The membranes were developed using Super Signal West Pico Chemiluminescent Substrate (Thermo Fisher Scientific Inc.) followed by exposure to a CCD camera (Luminescent image analyzer, GE Healthcare UK Ltd., Buckinghamshire, UK), and analyzed using Image quant LAS4000 software (GE Healthcare UK Ltd.).

### Active Rap-1 pull-down assay

Active Rap-1 was assessed using the Active Rap-1 Pull-Down and Detection Kit (Thermo Fisher Scientific Inc.) according to the manufacturer’s instructions. HAECs were starved overnight in EBM-2. After starvation, HAECs were treated with 30 μM cilostazol for 5 min and then lysed. The lysates (500 μg) were incubated with GST-RalGDS-RBD and Glutathione Resin. Samples were separated on a 4–20% SDS-PAGE and transferred to nitrocellulose membranes. After blocking with 0.5% blocking reagent (PerkinElmer Inc.) in PBS, the membranes were incubated overnight at 4°C with a rabbit monoclonal anti-Rap-1 antibody, followed by incubation with a peroxidase-conjugated goat anti-rabbit IgG (H+L) (dilution, 1:1000; Thermo Fisher Scientific Inc.). The membranes were developed using Super Signal West Pico Chemiluminescent Substrate (Thermo Fisher Scientific Inc.) followed by exposure to a CCD camera, and analyzed using Image quant LAS4000 (GE Healthcare UK Ltd.).

### Biacore experiments

All experiments were performed using Biacore S51 (GE Healthcare, Uppsala, Sweden) and carried out at 25°C with 5% DMSO-HBS-EP^+^ (GE Healthcare) used as a continuous flow buffer. Synthetic peptides of PDE3B corresponding to the domain interacting with Epac-1 or PI3Kγ at 2 mg/mL were immobilized on sensor chip SA (GE Healthcare). For immobilization, biotin-labeled Epac-1-binding PDE3B peptide or PI3Kγ peptide was injected in running buffer for 120 s at a flow rate of 10 μL/min. Final immobilization levels were between 850 and 1250 resonance units (RU). For the direct binding and competition assays, test drugs were injected in running buffer for 120 s, and then an undisturbed dissociation phase was monitored for 180 s at a flow rate of 30 μL/min. In the direct binding assay, test drags (0.3125–5 μmol/L) were injected alone. In the surface competition assays, test drags (0.625–5 μmol/L) were injected in the presence or absence of PDE3B-binding Epac-1 peptide-1 or PDE3B-binding Epac-1 peptide-2 (50 nmol/L). The rate constants of association and dissociation were calculated by BIA evaluation software (GE Healthcare UK Ltd.).

### Statistical Analysis

Values are expressed as the mean ± SEM of four to five experiments. Differences were considered statistically significant at *p* < 0.05. All analyses were performed with the Statistical Analysis System (SAS) software (Release 9.4, SAS).

## Results

### Functional expression of PDE3 isozymes in HAECs

**Fig 1:**
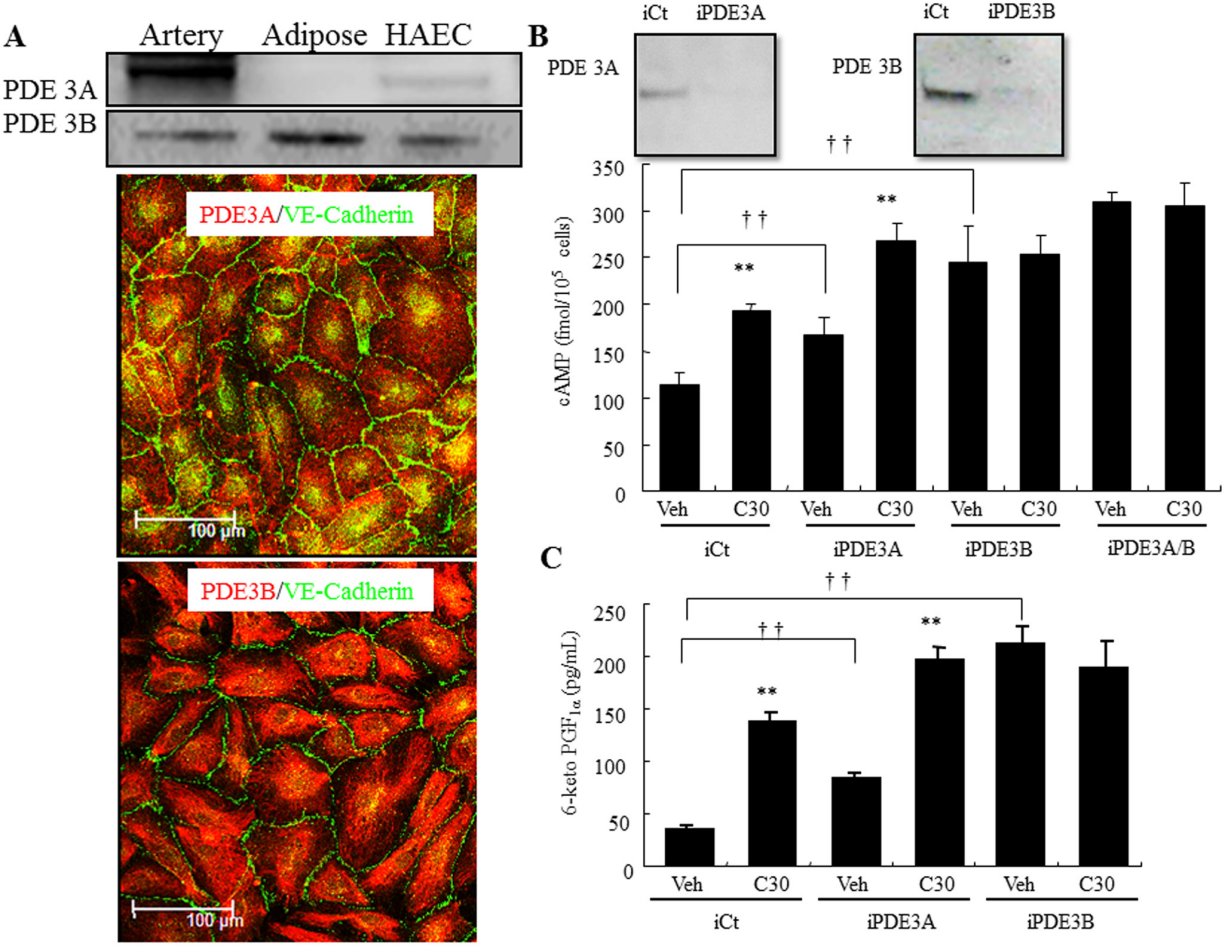
Functional expression of PDE3 in HAECs. (A) Top, Representative immunoblot showing PDE3A and PDE3B expression in human adipose, artery, and HAECs. Bottom, Fluorescence microscopy analysis of PDE3A and PDE3B expression in HAECs (green, VE-Cadherin; red, PDE3A or 3B). (B) Top, Effect of PDE3-targeting siRNAs (iPDE3A and 3B) or non-targeting siRNAs (iCt) on protein levels in HAEC. Bottom, Down-regulation of PDE3A and PDE3B expression increases intracellular cAMP levels in HAECs. HAECs were transfected with a iCT, iPDE3A, or iPDE3B. Post-transfection HAECs were treated with vehicle (Veh) or 30 μM cilostazol (C30), and then the intracellular cAMP concentration was determined. (C) Down-regulation of PDE3A and PDE3B expression increases PGI_2_ production in HAECs. HAECs were transfected with a iCT, iPDE3A, or iPDE3B. Post-transfection HAECs were treated with vehicle (Veh) or 30 μM cilostazol (C30), and then the PGI_2_ concentration in medium was determined (n = 4; †† *p* < 0.01 vs. iCT, *t*-test; ** *p* < 0.01 vs. Veh, *t*-test).

Western blot analysis and immunofluorescence microscopy showed that both PDE3 isoforms were expressed in HAECs, however, the PDE3A expression level was much lower than PDE3B (Fig 1A). Correspondingly, silencing either PDE3A or 3B elicited a significant increase in the basal level of intracellular cAMP level compared with the control siRNA-transfected cells, but the increased intracellular cAMP level was less in PDE3A-depleted cells than in PDE3B-depleted cells (1.7-fold and 2.4-fold, respectively; Fig 1B). Similar to the control siRNA-transfected cells, cilostazol (30 μM) significantly increased intracellular cAMP level in the PDE3A-depleted cells (1.73-fold and 1.57-fold respectively; Fig 1B), but not in the PDE3B-depleted cells. Silencing both PDE3A/B slightly increased intracellular cAMP level compared with PDE3A- or PDE3B-depleted cells.

### Cilostazol increases PGI_2_ production via the arachidonic acid cascade

**Fig 2:**
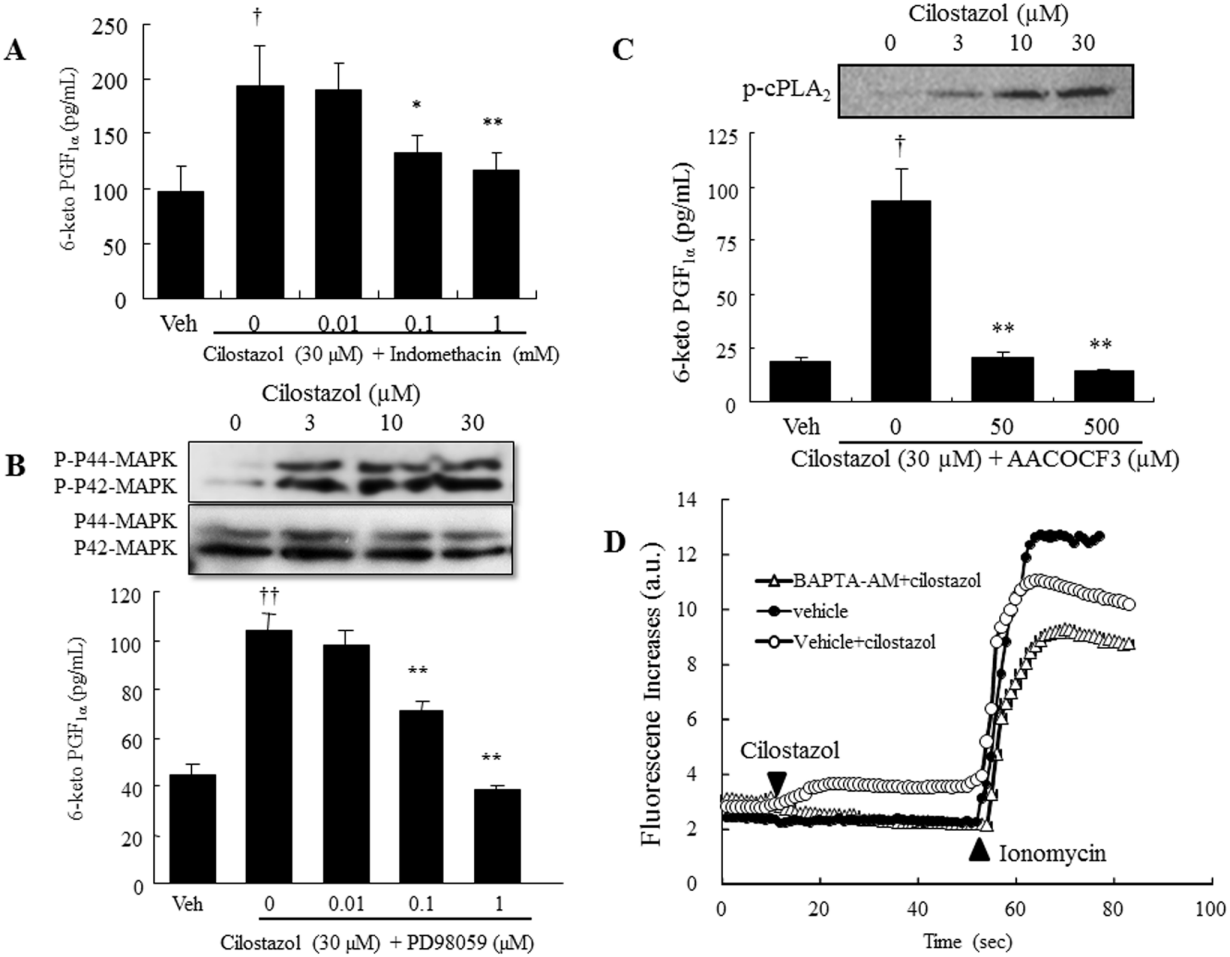
Cilostazol increases PGI_2_ production via the arachidonic acid cascade. (A) Effect of the COX inhibitor, indomethacin, on cilostazol-induced PGI_2_ production (n = 5; † *p* < 0.05 vs. vehicle, *t*-test; * *p* < 0.05, ** *p* < 0.01 vs. 30 μM cilostazol, lower-tailed Williams’ test). (B) Top, Representative immunoblot showing pMAPK and MAPK expression in HAECs. Bottom, Effect of the MEK inhibitor, PD98059, on PGI_2_ production in HAECs (n = 4, †† *p* < 0.01 vs. vehicle, *t*-test; ** *p* < 0.01 vs. 30 μM cilostazol *t*-test). (C) Top, Representative immunoblot showing p-cPLA_2_ (Ser-505) and total cPLA_2_ expression in HAEC. Bottom, Effect of AACOCF3 on cilostazol-induced PGI_2_ production (n = 5; † *p* < 0.05, vs. vehicle, *t*-test; ** *p* < 0.01 vs. 30 μM cilostazol, lower-tailed Williams’ test). (D) Effect of cilostazol on intracellular calcium levels in HAECs. Fluo-4-loaded HAECs were pretreated with 100 μM BAPTA-AM for 15 min, and then cells were treated with cilostazol (30 μM, 50 s) followed by stimulation with 1 μM ionomycin (n = 4).

Corresponding to intracellular cAMP levels, PGI_2_ release increased in both PDE3A- and PDE3B-depleted cells (1.75-fold and 5-fold respectively; Fig 1C). Treatment with cilostazol (30 μM) increased PGI_2_ production in the control siRNA-transfected cells by 3.6-fold and in the PDE3A-depleted cells by 2.5-fold, but not in the PDE3B-depleted cells (Fig 1C). Cilostazol-induced PGI_2_ production was significantly inhibited in a dose-dependent manner by the non-selective COX inhibitor, indomethacin, and was completely abolished at the concentration of 1 mM (Fig 2A). Mitogen-activated protein kinases (MAPKs) are key mediators of agonist-induced PGI_2_ production via direct phosphorylation of cPLA_2_α, resulting in the release of arachidonic acid [14]. Western blot analysis showed that cilostazol induced p42/44 MAPK phosphorylation without changing expression levels (Fig 2B). Furthermore, extracellular signal-regulated kinase (ERK) inhibitor completely abolished cilostazol-induced PGI_2_ production (10 μM; Fig 2B). Treatment with the cPLA_2_ inhibitor, AACOCF3 (50 μM), completely abolished cilostazol-induced PGI_2_ production (Fig 2C). The phosphorylation of cPLA_2α_ on Ser-505 was also enhanced by cilostazol in a dose-dependent manner without changing its total protein level.

### Cilostazol-induced PGI_2_ production is dependent on IP_3_ receptor-mediated intracellular calcium elevation

**Fig 3:**
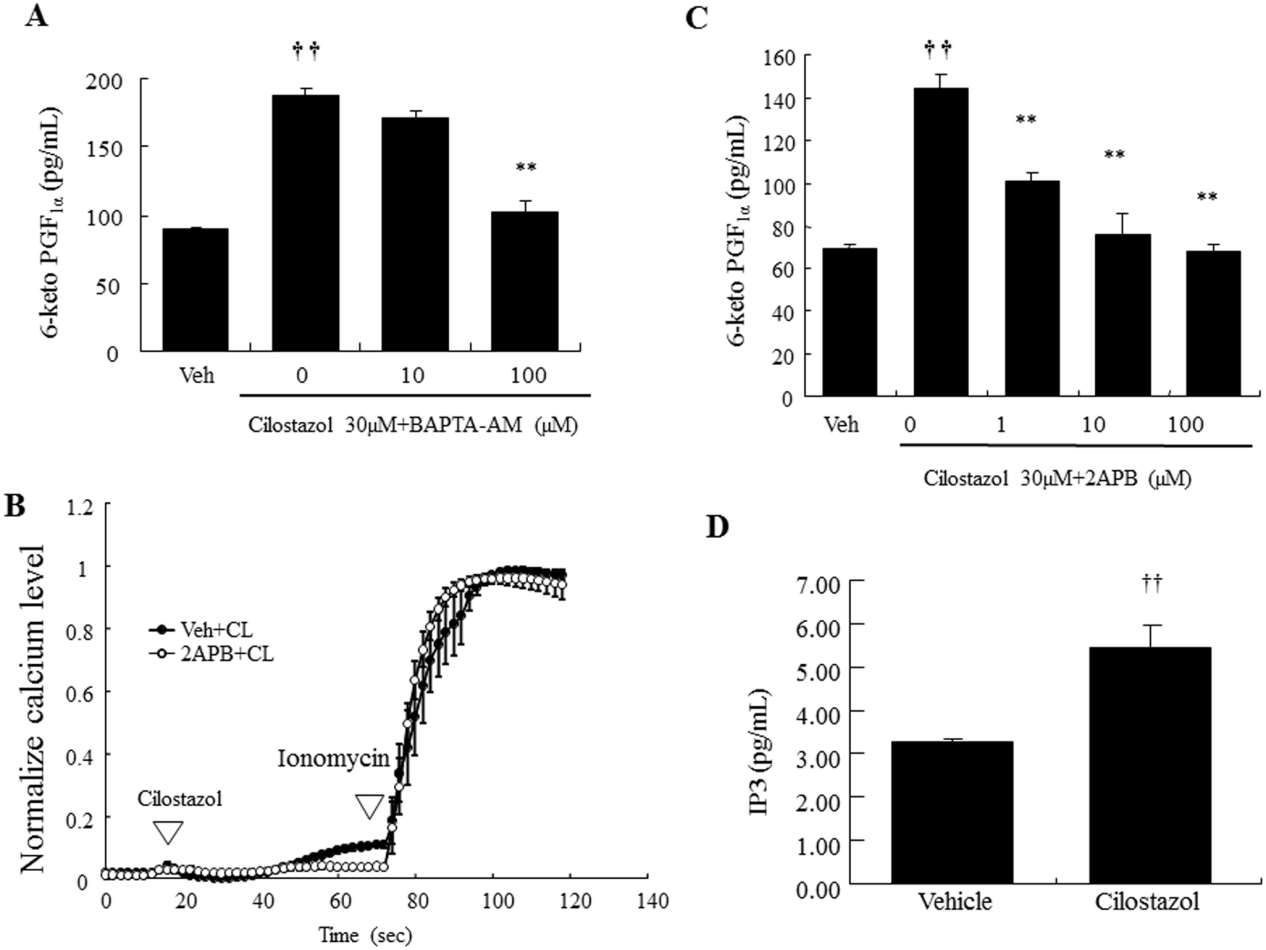
Cilostazol-induced PGI_2_ production is dependent on IP3 receptor-mediated intracellular calcium elevation. (A) Inhibitory effects of BAPTA-AM on cilostazol-induced PGI_2_ production (n = 5; †† *p* < 0.01 vs. vehicle, *t*-test; ** *p* < 0.01 vs. 30 μM cilostazol alone, lower-tailed Williams’ test). (B) Effect of 2-APB on cilostazol-induced intracellular calcium levels. Fluo-4-loaded HAEC were pretreated with vehicle, 2-APB (100 μM), for 15 min and then cells were treated with cilostazol (30 μM, 50 s) followed by stimulation with ionomycin (1 μM). Data were normalized against the maximal intensity obtained with 1 μM ionomycin. (C) Inhibitory effect of 2-APB on cilostazol-induced PGI_2_ production (n = 4; †† *p* < 0.01 vs. vehicle, *t*-test; ** *p* < 0.01 vs. 30 μM cilostazol, lower-tailed Williams’ test). (D) Effect of cilostazol (30 μM) on IP3 production (n = 4, †† *p* < 0.01 vs. vehicle, *t*-test).

The involvement of intracellular calcium elevation in cilostazol-induced PGI_2_ production was examined by chelating intracellular calcium. Fluo-4 fluorescence images of the intracellular calcium response in HAECs showed that fluorescence was immediately elevated by cilostazol stimulation (30 μM; Fig 2D). This increase was completely suppressed by BAPTA-AM (100μM). Consistently, cilostazol-induced PGI_2_ production was significantly decreased in a concentration-dependent manner using BAPTA-AM (Fig 3A), and 100 μM BAPTA-AM completely abolished cilostazol-induced PGI_2_ production. In addition, cilostazol-induced intracellular calcium elevation was almost completely abolished by the IP3R antagonist, 2-APB (100μM, Fig 3B). Consistent with inhibition of calcium elevation, 2-APB significantly inhibited PGI_2_ production in a dose-dependent manner, with complete inhibition at 100 μM (Fig 3C). In addition, cilostazol exerts a significant increase in IP3 levels (Fig 3D).

### Cilostazol induces PGI_2_ production in an Epac-1-dependent and PKA-independent manner

**Fig 4:**
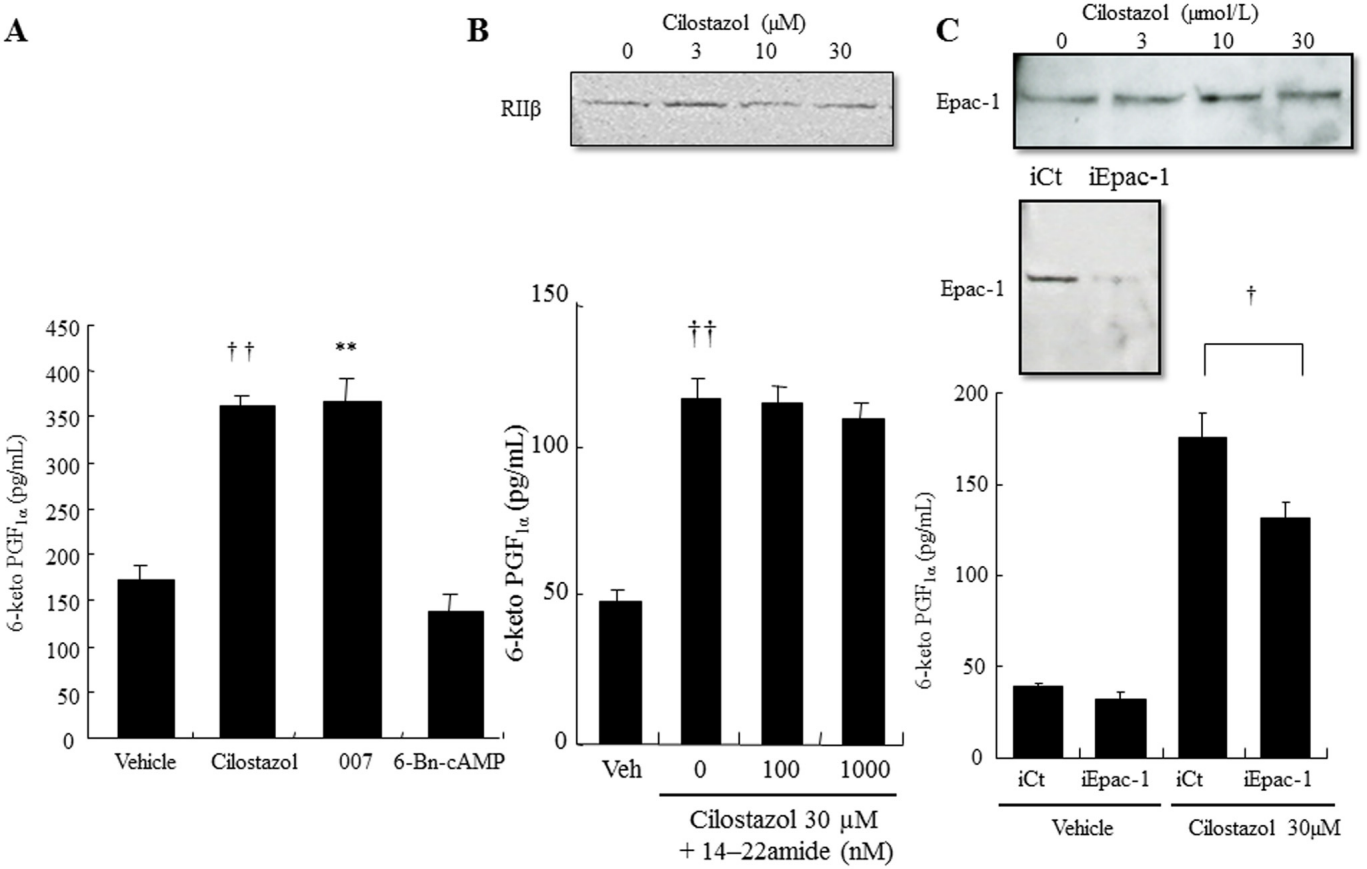
Epac-1 signaling, but not PKA, mediates cilostazol-induced PGI_2_ production. (A) Effect of cAMP effectors on PGI_2_ production. HAECs were treated with vehicle (0.05% DMSO), cilostazol (30 μM), 007 (10 μM), or 6-Bn-cAMP (n = 4; †† *p* < 0.01 vs. vehicle, ** *p* < 0.01 vs. cilostazol, *t*-test). (B) Top, Representative immunoblot showing total PKA type IIβ (RIIβ) expression in HAECs. Bottom, Effect of the PKA inhibitor, 14–22 amide, on PGI_2_ production in HAECs (n = 4; †† *p* < 0.01 vs. vehicle, *t*-test). (C) Top, Representative immunoblot showing total Epac-1 expression in HAECs. Middle, Effect of Epac-1-targeting siRNAs (iEpac-1) or non-targeting siRNAs (iCt) on protein levels in HAEC. Bottom, Effect of Epac-1 knockdown on PGI_2_ production in HAECs. HAECs were transfected with iEPAC-1, or with iCT. Post-transfection HAECs were treated with vehicle or 30 μM cilostazol (n = 4; † *p* < 0.05 vs. vehicle *t*-test).

The involvement of two major downstream effectors of cAMP, PKA and Epac-1, in cilostazol-induced PGI_2_ production was evaluated by the PKA-selective cAMP analogue, 6-Bn-cAMP, and the Epac-1-selective cAMP analogue, 007. 007 (100 μM) significantly increased PGI_2_ production to the same level as cilostazol (2-fold; Fig 4A), whereas 6-Bn-cAMP (100 μM) did not alter PGI_2_ levels (Fig 4A). The PKA inhibitor, 14–22 amide, did not affect the cilostazol-induced PGI_2_ production (Fig 4B lower panel), whereas silencing Epac-1 significantly decreased cilostazol-induced PGI_2_ production (30%; Fig 4C lower panel). Western blotting showed that cilostazol treatment did not alter protein levels of Epac-1 and PKA RIIβ (Fig 4B and 4C, lower panels).

### Epac-1 signaling pathways are involved in cilostazol-induced PGI_2_ production

**Fig 5:**
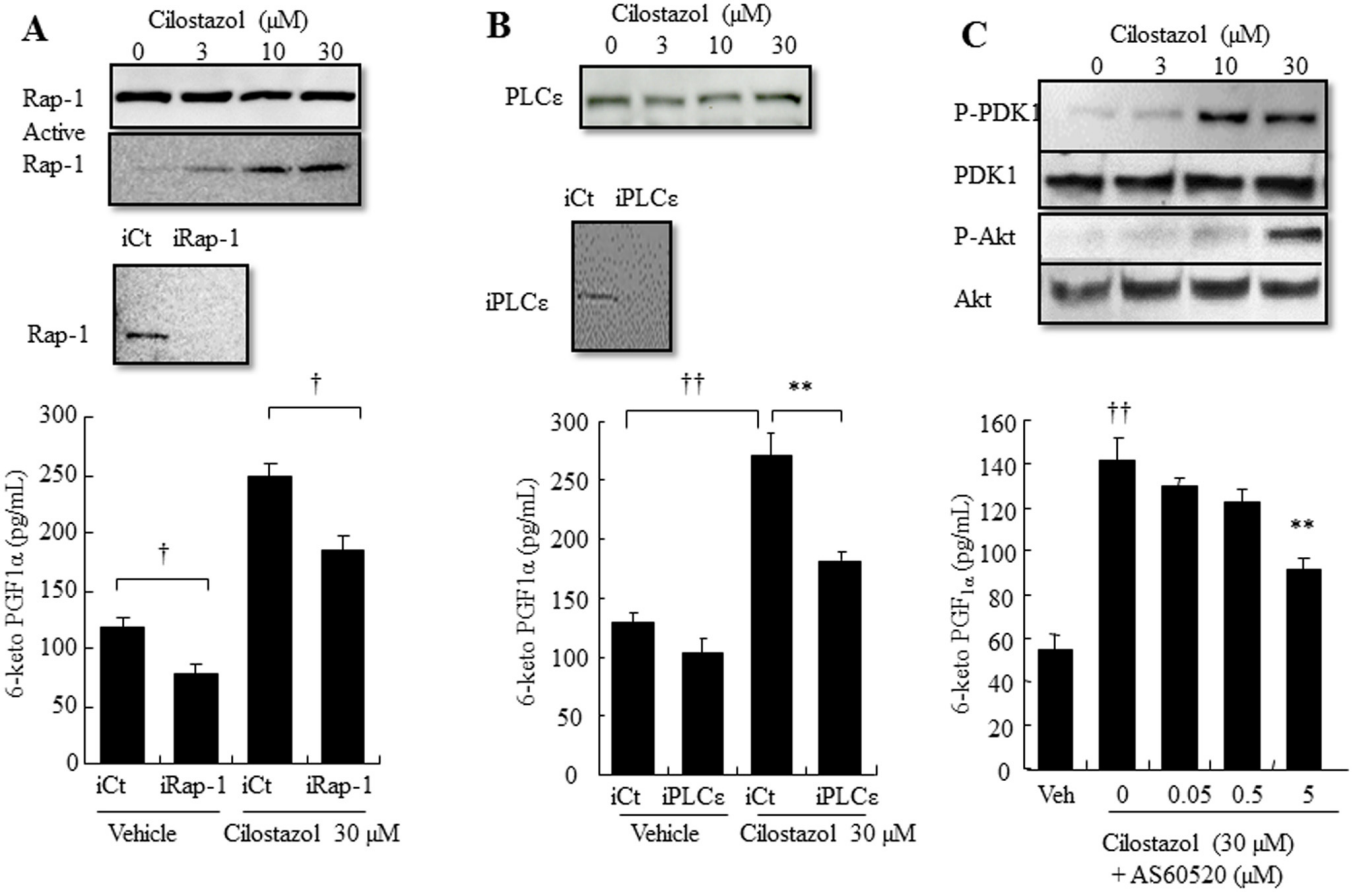
Epac-1/Rap-1 and its downstream signaling Akt and PLCε, but not Rac-1, mediate cilostazol-induced PGI_2_ production. (A) Top, Representative immunoblots showing total Rap-1 expression and Rap-1 activation in HAECs. Middle, Effect of Rap-1-targeting siRNAs (iRap-1) or non-targeting siRNAs (iCt) on protein levels in HAECs. Bottom, Effect of Rap-1 knockdown on PGI_2_ production in HAECs. HAECs were transfected with iRap-1, or with the iCT. Post-transfection HAECs were treated with vehicle or 30 μM cilostazol (n = 4; † *p* < 0.05 vs. vehicle *t*-test). (B) Top, Representative immunoblot showing PLCε expression in HAECs. Middle, Effect of PLCε-targeting siRNAs (iPLCε) or non-targeting siRNAs (iCt) on protein levels in HAECs. Bottom, Effect of PLCε knockdown on PGI_2_ production in HAECs. HAECs were transfected with iPLCε, or with the iCT. Post-transfection HAECs were treated with vehicle or 30 μM cilostazol (n = 4;† *p* < 0.05, †† *p* < 0.01 vs. iCT, *t*-test). (C) Top, Representative immunoblot showing P-PDK1, PDK1, Akt, and P-Akt expression in HAECs. Bottom, Effect of the PI3Kγ antagonist, AS60520, on PGI_2_ production in HAECs (n = 4, †† *p* < 0.01 vs. vehicle, *t*-test; ** *p* < 0.01 vs. 30 μM cilostazol alone, lower-tailed Williams’ test).

The major catalytic function of Epac-1 is a guanine nucleotide exchange that results in Rap-1 directly regulating the Rap-mediated downstream effectors, phospholipase C ε (PLCε) and ERK1/2, and indirectly regulating PKB [15]. The involvement of Epac-1/Rap-1 signaling in cilostazol-induced PGI_2_ production was evaluated by siRNA or an inhibitor. Silencing Rap-1 significantly decreased the basal level and cilostazol-induced PGI_2_ production (35% and 36%, respectively; Fig 5A lower panel). Western blotting showed that cilostazol treatment did not change protein expression of Rap-1, whereas the active Rap-1 pull-down assay showed that cilostazol activated Rap-1 in a dose-dependent manner (Fig 5A upper panel). Silencing PLCε significantly suppressed cilostazol-induced PGI_2_ production and slightly suppressed the basal level of PGI_2_ production (38% and 20% respectively; Fig 5B lower panel). Similar to Epac-1 and Rap-1 siRNA-depleted cells, cilostazo did not change total protein expression level (Fig 5B upper panel). Exposure of HAECs to the selective PI3Kγ inhibitor, AS60520, caused a significant concentration-dependent decrease in cilostazol-induced PGI_2_ production (42%; Fig 5C lower panel). Consistently, cilostazol increased phosphorylation of 3-phosphoinositide-dependent protein kinase 1 (PDK1) and Akt, which are downstream effectors of PI3K (Fig 5C upper panel). In addition, silencing Epac-1 decreased cilostazol-induced ERK and MAPK, as well as Akt phosphorylation (S1 Fig).

### Cilostazol and other cAMP-elevating agents affect PGI_2_ production differently

**Fig 6:**
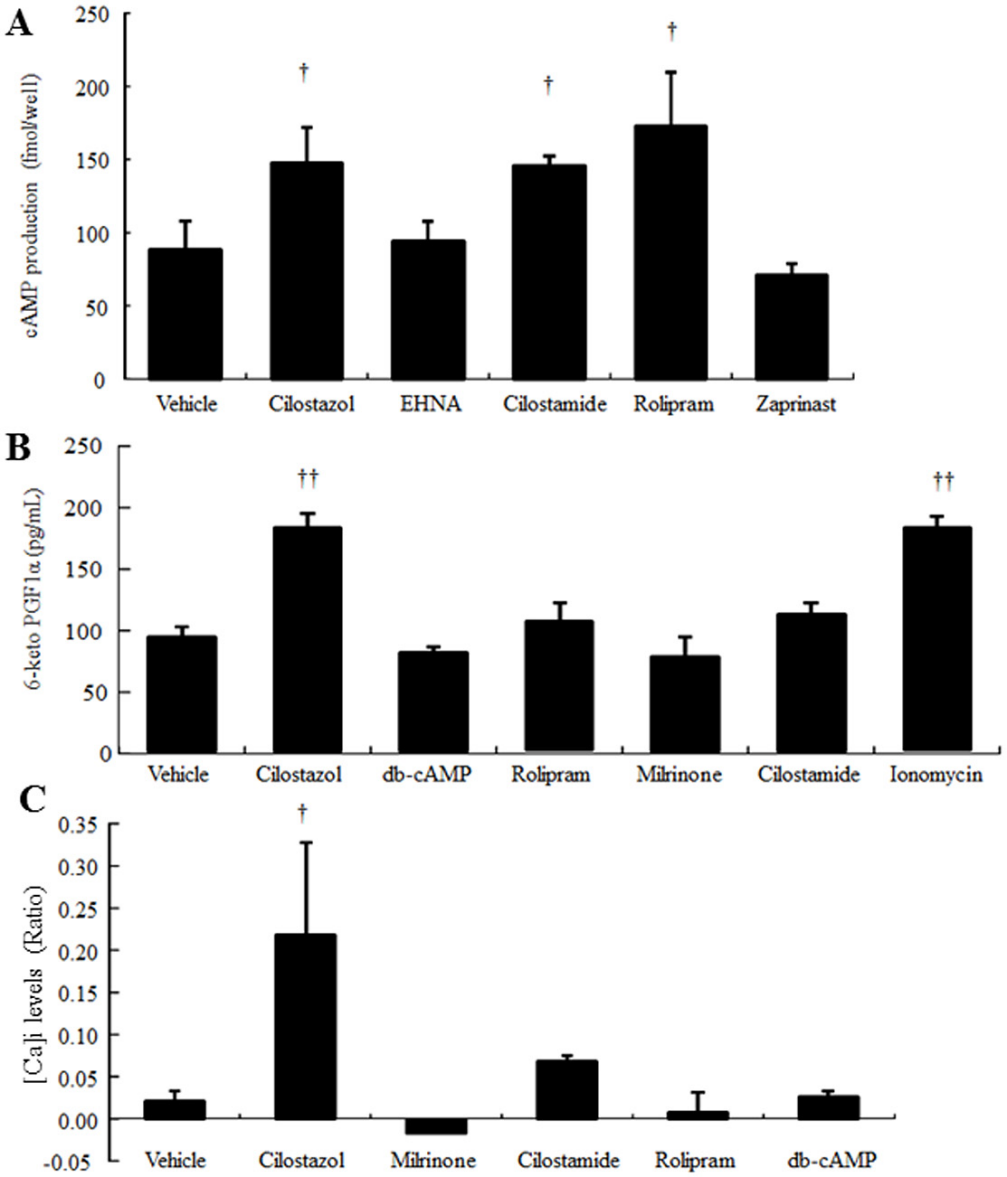
Different cAMP-elevating drugs affect cilostazol-induced PGI_2_ production differently in HAECs. (A) Effect of cAMP-elevating agents on intracellular cAMP levels. HAECs were treated with vehicle (0.05% DMSO), cilostazol (30 μM), EHNA (100 μM), cilostamide (30 μM), rolipram (10 μM), or zaprinast (50 μM) (n = 4; † *p* < 0.05 vs. vehicle, *t*-test). (B) Effect of cAMP-elevating agents on PGI_2_ production. HAECs were treated with vehicle (0.05% DMSO), cilostazol (30 μM), db-cAMP (100 μM), rolipram (10 μM), milrinone (30 μM), cilostamide (30 μM), or ionomycin (1 μM) (n = 4; ** *p* < 0.01 vs. vehicle, *t*-test). (C) Effect of cAMP-elevating agents on intracellular calcium levels in HAECs. Fluo-4-loaded HAECs were treated with vehicle (0.05% DMSO), cilostazol (30 μM), db-cAMP (100 μM), rolipram (10 μM), milrinone (30 μM), or cilostamide (30 μM) for 50 s followed by stimulation with 1 μM ionomycin. Data were normalized against the maximal intensity obtained with 1 μM ionomycin (n = 4, †*p* < 0.05 vs. vehicle, randomized Dunnett’s test).

Next, we characterized the effects of other cAMP-elevating agents on PGI_2_ production. We found that cilostamide (30 μM) and rolipram (10 μM) significantly increased intracellular cAMP expression (1.65- and 1.96-fold, respectively; Fig 6A). Like cilostamide, cilostazol (30 μM) showed a 1.68-fold increase in intracellular cAMP expression (Fig 6A). Moreover, cilostazol significantly increased PGI_2_ production (1.95-fold, Fig 6B). Cilostamide only slightly increased PGI_2_ production (1.2-fold, Fig 6B). In contrast, db-cAMP (100 μM) and milrinone slightly decreased PGI_2_ expression (Fig 6B). Intracellular calcium level was decreased by milrinone (100 μM), rolipram, and db-cAMP, whereas cilostamide increased the intracellular calcium level (3.4-fold, Fig 6C). Cilostazol (30 μM) significantly increased the intracellular calcium level by 10.36-fold (Fig 6C).

### Cilostazol directly interacts with Epac-1-binding PDE3B peptide

**Fig 7:**
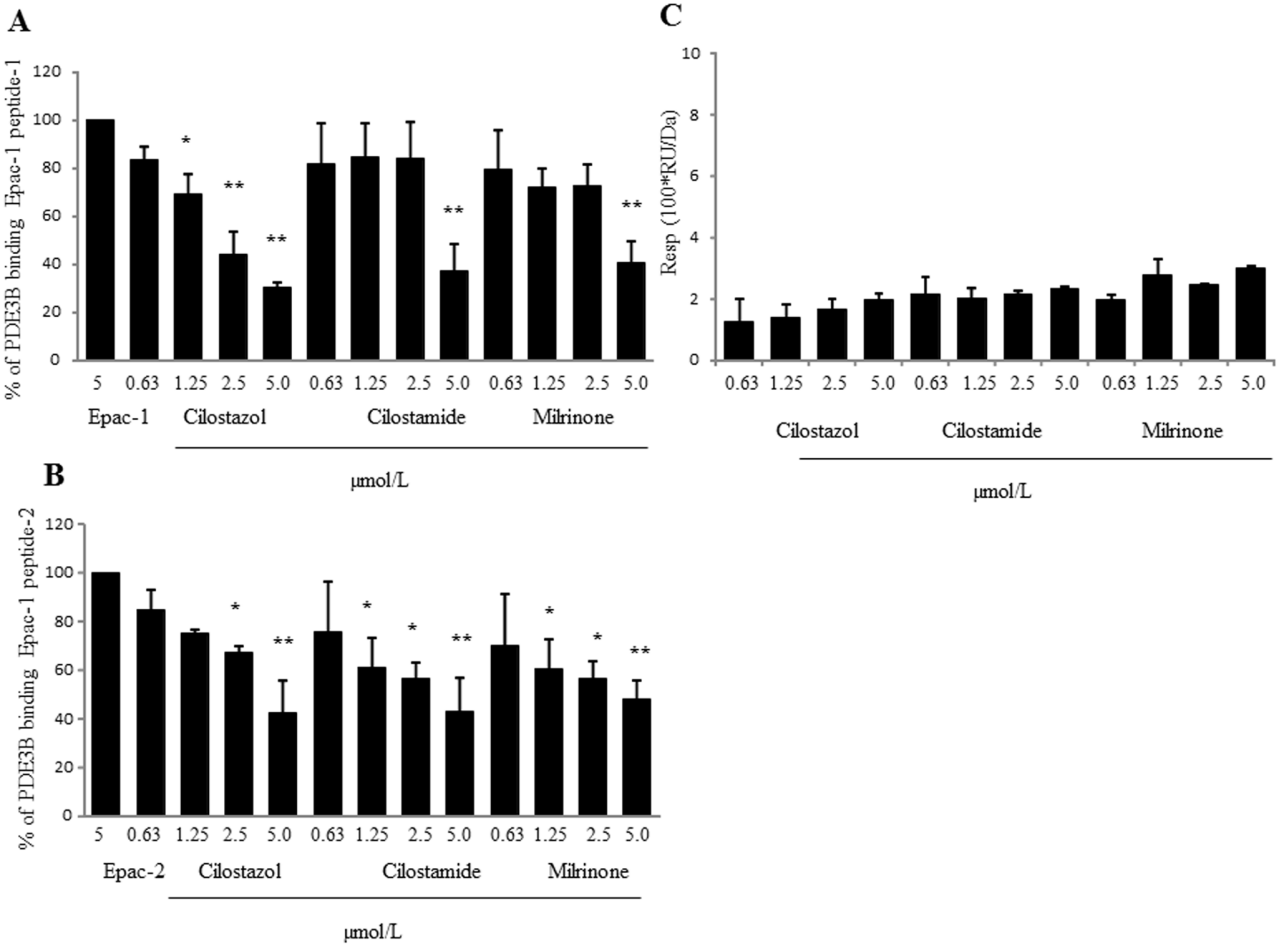
Biocore analysis of cilostazol-PDE3B/Epac-1 interaction. (A) Cilostazol, cilostamide, and milrinone interfere with association of PDE3B-binding Epac-1 peptides-1 to PDE3B. The above-mentioned compounds’ competition with PDE3B-binding Epac-1 peptides-1 (5 μM) binding to Epac-1-binding PDE3B peptide was evaluated (n = 4; ** *p* < 0.01 vs. 5 μM PDE3B-binding Epac-1 peptides-1, randomized Dunnett’s test). (B) Cilostazol, cilostamide, and milrinone interfere with association of PDE3B-binding Epac-1 peptides-2 to PDE3B. These compounds’ competition with Epac-1 peptides-2 (5 μM) binding to Epac-1-binding PDE3B peptide was evaluated (n = 4; * *p* < 0.05, ** *p* < 0.01 vs. 5 μM PDE3B-binding Epac-1 peptides-2, randomized Dunnett’s test). (C) Direct bindings of cilostazol, cilostamide, and milrinone to PI3Kγ-binding PDE3B peptide. Relative responses of PI3Kγ-binding PDE3B peptide to drugs at concentrations of 0.3125, 0.625, 1.25, 2.5, and 5 μM (n = 4).

In the competitive binding analysis using Epac-1-binding with PDE3B peptide immobilized on a sensorchip, competitive binding of cilostazol, cilostamide, or milrinone with two PDE3B-binding Epac-1 peptides (5 μM) was evaluated. Cilostazol, cilostamide, and milrinone significantly inhibited the association of PDE3B-binding Epac-1 peptide-1 to Epac-1-binding PDE3B peptide in a dose-dependent manner, with maximal inhibition of 70%, 63%, and 60%, respectively (Fig 7A). Likewise, cilostazol, cilostamide, and milrinone significantly inhibited association of PDE3B-binding Epac-1 peptide-2 to Epac-1-binding PDE3B peptide in a dose-dependent manner, with maximal inhibitions of 58%, 57%, and 52%, respectively (Fig 7B). In contrast, 007 showed no affinity for PDE3B-binding Epac-1 peptide (S2 Fig). Additionally, 007 did not interfere with the association of PDE3B-binding Epac-1 peptides to Epac-1-binding PDE3B peptide and the results were below the detection limit. In contrast, in the direct binding assay using PI3Kγ-binding PDE3B peptide immobilized on a sensorchip, the response levels of the test compounds were very low, with RU values between 1.25 and 3.0 (Fig 7C). Thus, the competitive binding analysis using PI3Kγ-binding PDE3B peptide for test compounds was not evaluated.

## Discussion

**Fig 8:**
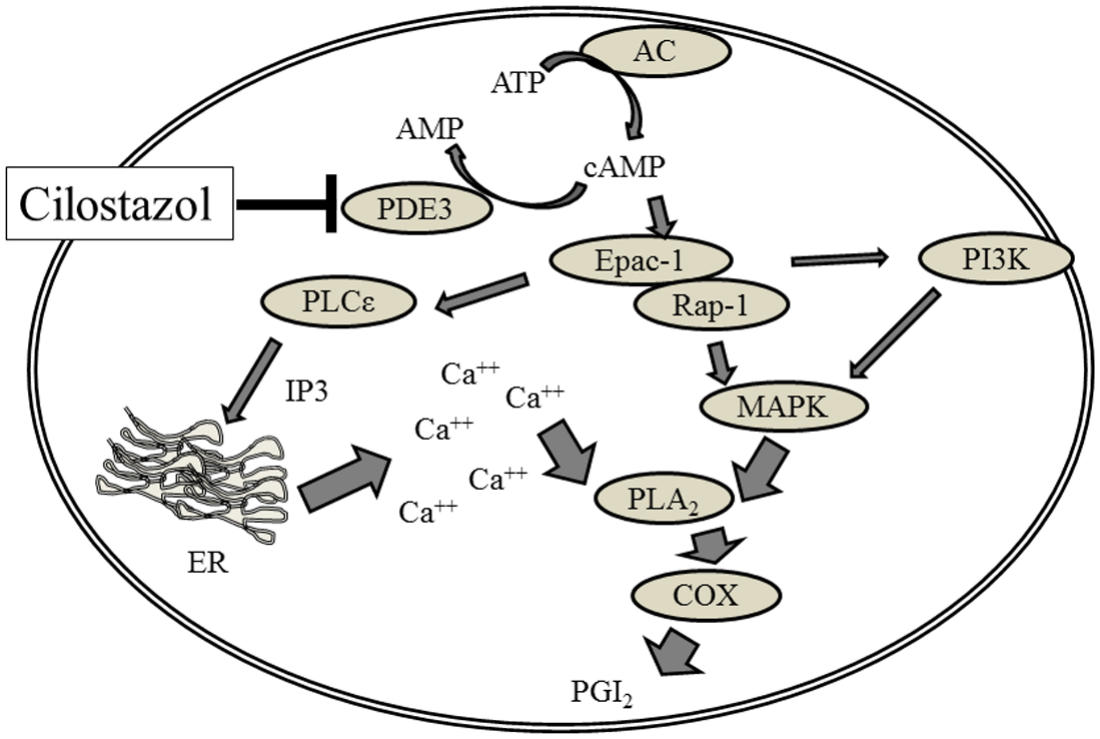
Schematic overview of cilostazo-induced PGI_2_ production in HAECs. Cilostzol-induced MAPK activation, combined with intracellular calcium elevation, results in PGI_2_ production in HAECs. Calcium elevation is triggered by inositol 3,4,5 triphosphate (IP3)-regulated calcium channels (IP3R) to activate cPLA2/COX signaling. In contrast, MAPK also plays a crucial role in cPLA2 activation. Activation of Epac-1signaling modulates both processes. PDE3 is partially involved in these processes.

The current study demonstrated the effect of cilostazol on PGI_2_ production and its mechanism in endothelial cells (Fig 8). We report several novel findings: first, cilostazol increases PGI_2_ synthesis in endothelial cells by activating arachidonic acid metabolism via the COX/PGI_2_ pathway. Second, the Epac-1/Rap-1, but not the PKA, signaling pathway is involved in cilostazol-induced PGI_2_ production. Third, the mechanism of cilostazol-induced PGI_2_ production involves increased intracellular calcium by releasing calcium from calcium stores via activation of the Epac-1/Rap-1/PLCε/IP3R pathway. Fourth, the Epac-1/Rap-1 mechanism of cilostazol directly activates MAPK and indirectly activates PI3Kγ. Because cilostazol is a potent PDE3 inhibitor (the IC_50_ values of PDE3A and PDE3B are 0.20 and 0.38 μM, respectively) [3] and PDE3s are expressed in HAECs, we initially predicted that intracellular cAMP accumulation is involved in cilostazol-induced PGI_2_ production. Indeed, under our experimental conditions, cilostazol increased both cAMP levels and PGI_2_ synthesis. Corresponding to expression levels and cAMP-catalyzing activities, PDE3B acts predominantly on PGI_2_ production. Thus, it seems reasonable to speculate that intracellular cAMP elevation is involved in the mechanism of cilostazol-induced PGI_2_ production in the endothelium. Downstream functions of cAMP are mediated by PKA and Epac. PKA provided a link between stimulation of adenylyl cyclase, and Epac acts as a cAMP-activated guanine nucleotide exchange factor for Rap [15]. Interestingly, pharmacological activation or inhibition of PKA showed no impact on the basal level or cilostazol-induced PGI_2_ production. In contrast, pharmacological activation and/or siRNA-mediated silencing of Epac-1/Rap-1 revealed that inhibition of Epac-1/Rap-1 signaling only partially suppressed cilostazol-induced PGI_2_ production, as the maximal inhibitions were only 30% and 36%, respectively. Furthermore, PI3K inhibition suppressed cilostazol-induced PGI_2_ production to the same extent as inhibition of Epac-1/Rap-1 did, with a maximal inhibition of 42%. The finding that HDL-induced COX-2 expression and PGI_2_ production were abolished by PI3K inhibitor in ECV304 endothelial cells with a maximal inhibition of 40% [16] supports our findings showing PI3K-mediated PGI_2_ production in endothelial cells. Indeed, non-selective COX inhibitor, indomethacin, with an IC_50_ for COX-1 and COX-2 of 0.063 μM and 0.48 μM, respectively [17], completely abolished cilostazol-induced PGI_2_ production in HAECs. In endothelial cells, COX-1 and COX-2 are constitutively expressed [18]. Thus, it seems reasonable to suggest that cilostazol promotes PGI_2_ production by activating COX-1 and COX-2 in HAEC. Further, Epac-1/Rap-1/PI3K signaling plays an important role in cilostazol-induced PGI_2_ production. In endothelial cells, calcium is essential for PGI_2_ synthesis [19]. That is, PGI_2_ synthesis is initiated by catalyzing the cleavage of arachidonic acid from membrane-bound lipids via cPLA_2_ activation depending on the intracellular calcium level [7]. A recent review on the physiological action of Epac [20] described new evidence showing that Epac directly interacts with intracellular calcium release channels, such as IP3 receptors via Rap/PLCε. In human dermal microvascular endothelial cells (HMEC-1), β2-adrenoceptor activation induces machinery that mobilizes intracellular calcium elevation via the G-protein/adenylyl cyclase/cAMP/Epac-1/IP3 pathway [21]. Correspondingly, we observed that inhibition of PLCε affected cilostazol-induced PGI_2_ production similarly to Epac-1/Rap1 inhibition with a maximal inhibition of 38%. Moreover, cilostazol increased intracellular calcium levels and IP3 release. Taken together, the mechanism of cilostazol-induced PGI_2_ production is mediated by intracellular calcium via Epac-1/Rap-1/PLCε/IP3R activation. In contrast, MAPK also plays an important role in cPLA_2_ activation by phosphorylating Ser-505, which acts synergistically with calcium to generate arachidonic acid [22], [23]. Contrary to Epac-1/Rap-1 and other downstream signaling inhibition, ERK1/2 inhibitor decreased cilostazol-induced PGI_2_ production to a basal level, suggesting that MAPK signaling pathway plays a major role in cilostazol-induced PGI_2_ production, and MAPK-mediated cilostazol-induced PGI_2_ production could not be explained by Epac-1/Rap-1 signaling. Recently, Wilson et al [24]. discovered the novel signaling complex, PDE3B-tethered EPAC1/p84-p110γ, which regulates Epac-1 binding to cAMP and PI3Kγ downstream signals, such as ERK and PKB, in HAECs. We previously demonstrated that cilostazol induced PKB phosphorylation, which was abolished by the wide-range PI3K inhibitor, LY294002, in HAECs [25]. The present study also demonstrated that cilostazol induced PDK, PKB, and MAPK phosphorylation in HAECs. These observations suggest that Epac-1/Rap-1 and Epac-1/PI3K signaling synergistically activate MAPK to generate PGI_2_. However, the Biacore analysis provided evidence showing that cilostazol and other PDE3 inhibitors directly binds to the Epac-1-binging domain of PDE3B and interferes with formation of the PDE3B-Epac-1 complex in a similar fashion. Furthermore, none of the PDE3 inhibitors blocked formation of the PDE3B-PI3K complex. Moreover, Epac-1 activator, 007, showed no affinity for the PDE3B binding region of Epac-1. Nevertheless, 007 strongly induced PGI2 production. Taken together, it seems that the PDE3B/Epac-1/PI3K complex plays a minor role in PGI_2_ production. Additionally, intracellular cAMP elevation has no impact on PGI_2_ production. Addition of other PDE inhibitors or db-cAMP did not increase PGI_2_ production. Our observations are consistent with earlier reports showing no correlation between global cAMP levels and PGI_2_ synthesis in endothelial cells [26]. Milrinone, another PDE3 inhibitor that is structurally unrelated to cilostazol with a similar PDE3 inhibition potency [27], slightly decreased intracellular calcium levels and PGI_2_. In contrast, cilostamide slightly increased intracellular calcium levels and PGI_2_. We already mentioned that intracellular calcium elevation is essential for PGI_2_ synthesis, and differences in PGI_2_ synthesis by cAMP-elevating agents are likely due to differences in regulation of intracellular calcium elevation. These findings support our hypothesis that crosstalk between multiple signaling pathways initiates intracellular calcium elevation and MAPK activation via cilostazol. Hence, intracellular cAMP accumulation seems necessary, though it has a minor function in cilostazol-induced PGI_2_ synthesis, and it appears that mechanisms other than cAMP accumulation also contribute to cilostazol-induced PGI_2_ synthesis. Because cilostazol is also an adenosine uptake inhibitor, this indicates the possible existence of crosstalk between the Epac-1/Rap-1 pathway and the signaling cascade from adenosine receptors to extracellular adenosine elevation, to produce PGI_2_. Recent evidence supports our hypothesis that adenosine-mediated signaling is involved in prostaglandin synthesis in the endothelium and thatactivation of adenosine A1 receptor increases PGI_2_ synthesis in the rat aorta [28] and rat aortic endothelial cells [28], [29]. Furthermore, milrinone is also known as an adenosine A1 receptor antagonist [30]. Therefore, it would be reasonable to speculate that milrinone inhibits the adenosine A1 receptor, thereby decreasing PGI_2_ production. The involvement of adenosine receptor activation in cilostazol-induced PGI_2_ production is now under consideration. However in this study, we concluded that HAEC stimulation with cilostazol induces increased intracellular calcium by activating calcium release from intracellular calcium stores via IP3 receptor activation, along with Epac-1/Rap-1/PLCε and Epac-1/Rap-1/MAPK activation, resulting in a synergistic increase in PGI_2_ production. These results provide new evidence showing that the PDE3B/Epac-1 signaling pathway mediates cilostazol-induced PGI_2_ release from HAECs via an increase in intracellular calcium.

## Supporting Information

S1 Fig Epac-1 mediates cilostazol-induced MAPK and Akt in HAECs. (A) **Top**, Effect of Epac-1-targeting siRNAs (iEpac-1) or non-targeting siRNAs (iCt) on phosphorylation of MAPK and Akt in HAECs. **Bottom**, Effect of Epac-1-targeting siRNAs (iEpac-1) or non-targeting siRNAs (iCt) on cilostazol-induced phosphorylation of ERK and Akt in HAECs. HAECs were transfected with iEPAC-1, or with iCT. Post-transfection HAECs were treated with vehicle or 30 μM cilostazol (n = 4; * *p* < 0.01 vs. iCT, *t*-test). Phosphorylation of both proteins was normalized with their total proteins.
(PPTX)

S2 Fig Biocore analysis of Epac-1 activator (007)-PDE3B/Epac-1 interaction. Direct bindings of 007 to Epac-1-binding PDE3B peptide. Relative responses of Epac-1-binding PDE3B peptide to 007 at concentrations of 1.25, 2.5, and 5μM. Cilostazol (5 μM, CL5) was used as a positive control (n = 4).
(PPTX)
