# Supplementary material for: Cilostazol Induces PGI2 Production via Activation of the Downstream Epac-1/Rap1 Signaling Cascade to Increase Intracellular Calcium by PLCε and to Activate p44/42 MAPK in Human Aortic Endothelial Cells
Source: PLoS One. 2015 Jul 16;10(7):e0132835. doi: 10.1371/journal.pone.0132835 (PMC4504471; doi:10.1371/journal.pone.0132835)
Supplement: S1 Fig — (A) Top, Effect of Epac-1-targeting siRNAs (iEpac-1) or non-targeting siRNAs (iCt) on phosphorylation of MAPK and Akt in HAECs. Bottom, Effect of Epac-1-targeting siRNAs (iEpac-1) or non-targeting siRNAs (iCt) on cilostazol-induced phosphorylation of ERK and Akt in HAECs. HAECs were transfected with iEPAC-1, or with iCT. Post-transfection HAECs were treated with vehicle or 30 μM cilostazol (n = 4; * p < 0.01 vs. iCT, t-test). Phosphorylation of both proteins was normalized with their total proteins. (PPTX) [file pone.0132835.s001.pptx]

## Slide 1
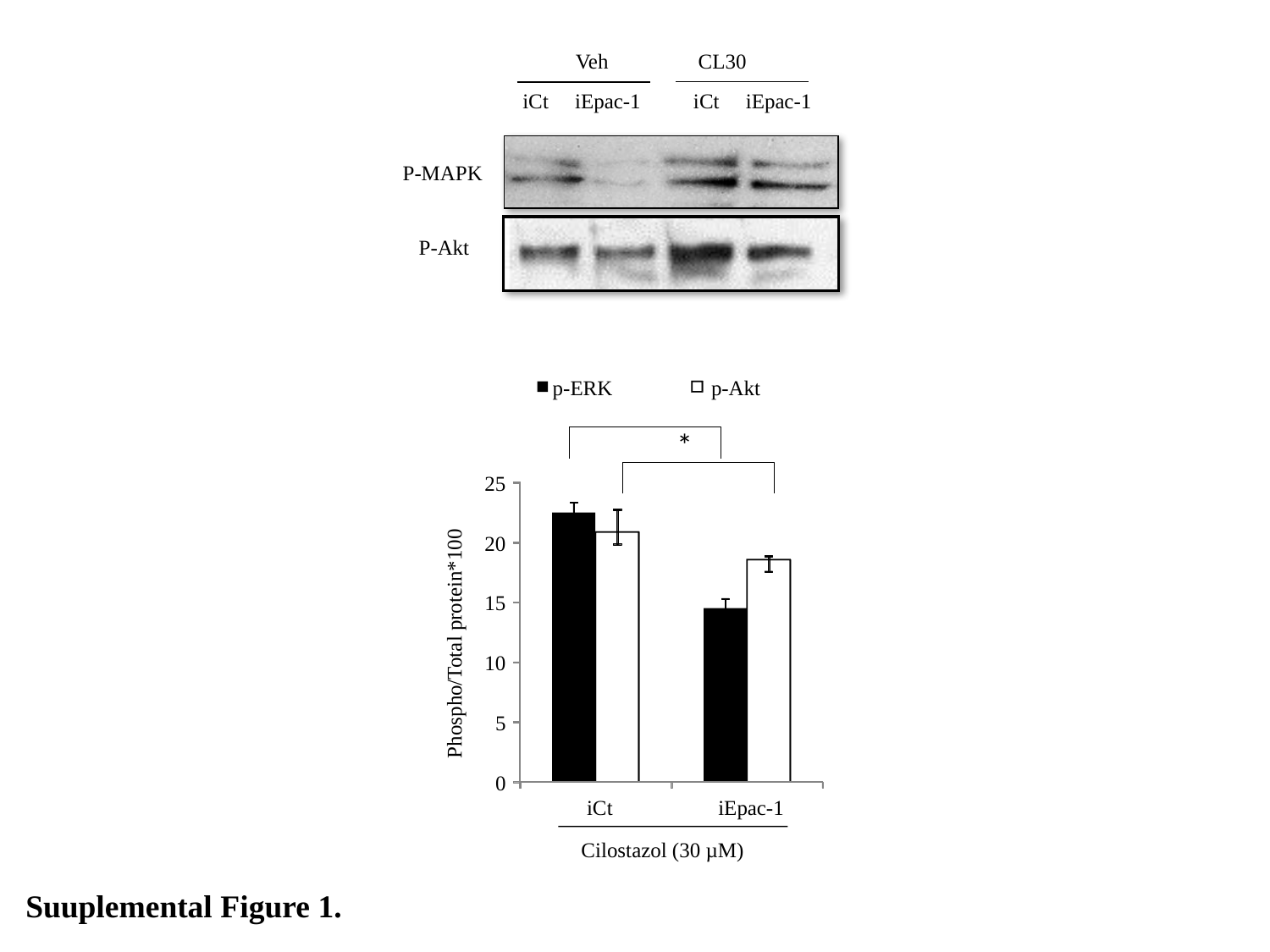

Veh CL30
 iCt iEpac-1 iCt iEpac-1
P-MAPK
P-Akt
p-ERK
p-Akt
*
*
25
20
15
Phospho/Total protein*100
10
5
0
iCt
iEpac-1
Cilostazol (30 µM)
Suuplemental Figure 1.
