# Supplementary figures and images for: Cilostazol Induces PGI2 Production via Activation of the Downstream Epac-1/Rap1 Signaling Cascade to Increase Intracellular Calcium by PLCε and to Activate p44/42 MAPK in Human Aortic Endothelial Cells
Source: PLoS One. 2015 Jul 16;10(7):e0132835. doi: 10.1371/journal.pone.0132835 (PMC4504471; doi:10.1371/journal.pone.0132835)

## Slide 1
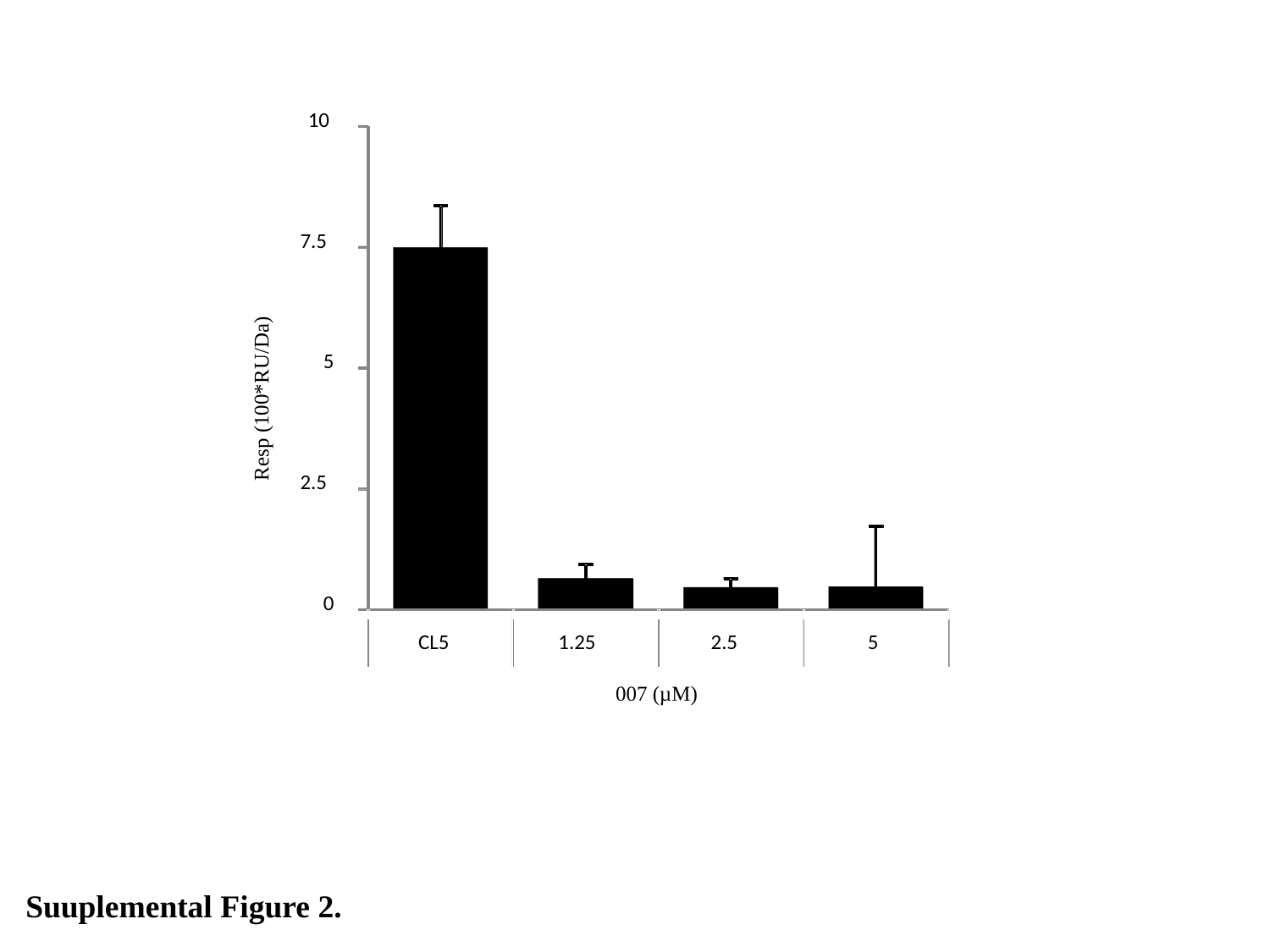

10
7.5
5
Resp (100*RU/Da)
2.5
0
CL5
1.25
2.5
5
007 (µM)
Suuplemental Figure 2.

Supplement: S2 Fig — Direct bindings of 007 to Epac-1-binding PDE3B peptide. Relative responses of Epac-1-binding PDE3B peptide to 007 at concentrations of 1.25, 2.5, and 5μM. Cilostazol (5 μM, CL5) was used as a positive control (n = 4). (PPTX) [file pone.0132835.s002.pptx]
